# COVID-19, AI enthusiasts, and toy datasets: radiology without radiologists

**DOI:** 10.1007/s00330-020-07453-w

**Published:** 2020-11-11

**Authors:** H. R. Tizhoosh, Jennifer Fratesi

**Affiliations:** 1 Kimia Lab, University of Waterloo, Waterloo, Canada; 2 Vector Institute, MaRS Centre, Toronto, Canada; 3 Department of Medical Imaging, University Health Network, Toronto, Canada

In computer science, textbooks talk about the “garbage in, garbage out” concept (GIGO); i.e., low-quality input data generates unreliable output or “garbage.” GIGO becomes, even more, a pressing issue when we are dealing with highly complex data modalities, such as radiographs and computed tomography scans.

The performance of any deep network directly depends on the quality of the dataset that it learns from. Reputable repositories like Cancer Imaging Archive [1] backed up with a large body of work by experts [2] is an example of reliable datasets. Adhering to DICOM standards and ensuring that images are properly linked to supporting metadata are obligatory to construct a well-curated dataset.

In recent weeks, we are observing a trend to hastily use ill-curated data to train deep networks for COVID-19. It seems AI enthusiasts impatiently create their own datasets of medical images without seeking clinical collaborators to guide them. These collections are rather “toy sets” through the manual gathering of publicly accessible images (e.g., online journals, and preprints on non-peer-reviewed archives). Most of the time AI researchers—with no clinical or medical competency—create their own experimental “toy” datasets to run initial investigations and establish a framework for algorithmic challenges.

To be clear, a “toy dataset” from the medical imaging perspective is not a toy just because it is very small and does not comply with DICOM standards, but more importantly because it has been created by engineers and computer scientists, and not by physicians and medical/clinical experts. Such datasets of COVID-19 images have been emerging on the Internet and used by AI enthusiasts to write blogs and non-peer-reviewed reports [3–7]. The training of the so-called COVID Nets happens with these toy datasets with no radiologist participation, and with no common validations such as “leave-one-out” testing. In an attempt to overcome the small data size, AI enthusiasts mix the few adult COVID-19 images scraped from the Internet with many pediatric (bacterial) pneumonia images [5, 6]; Are these COVID Nets learning anything meaningful?

No one can curate a COVID-19 dataset in disregard of professional recommendations. The American College of Radiology (ACR) and Canadian Association of Radiology (CAR) currently do not recommend the use of x-ray or CT imaging to screen or diagnose COVID-19 infections [8] because of risks for spreading the infection, resource constraints, and added logistics. However, CT, in particular, may be useful to expedite care in symptomatic patients with a negative or pending swab, and in those developing complications such as acute respiratory distress syndrome, and findings suspicious for COVID-19 are commonly being seen in high-risk patients incidentally. Findings on CT are non-specific and can overlap with other types of viral infections (such as influenza) and other non-infectious diseases, for example, organizing pneumonia and drug reaction but there are some characteristic features [9] and standardized reporting has been recently introduced by the RSNA [10]. A well-curated dataset should consider multiple phases:

- Early phase (2–4 days): bilateral, ground-glass opacities, rounded or nodular appearance (50%), peripheral and basal in distribution
- Intermediate phase (4–7 days): consolidation, reverse halo, crazy paving
- Late phase: consolidation, diffuse bilateral ground-glass opacities, organized pneumonia appearance

Faulty results based on creating amateur datasets and training sketchy AI solutions hastily to publish online may not make it to mainstream radiology due to the barriers of peer review; it may, however, create false hope among patients and patient advocacy groups, falsify the perception of government funding agencies and healthcare policy organizations, and misguide young scientists and resident radiologists. It is the duty of both serious AI researchers and expert radiologists to set the records straight: Any dataset of radiological images must be assembled by the participation of expert radiologists; there is no radiology without radiologists. Serious scientists have indeed recognized this and are delivering peer-reviewed papers using carefully curated image data [11, 12].
